# Comparative Analysis of Extracellular Vesicle and Virus Co-Purified Fractions Produced by Contemporary Influenza A and B Viruses in Different Human Cell Lines

**DOI:** 10.3390/v17111470

**Published:** 2025-11-04

**Authors:** Aude Wantchecon, Julien Boucher, Henintsoa Rabezanahary, Caroline Gilbert, Mariana Baz

**Affiliations:** 1 Axe de Recherche Maladies Infectieuses et Immunitaires, Centre de Recherche du CHU de Québec-Université Laval, Quebec City, QC G1V 4G2, Canada; aude.wantchecon-croix@crchudequebec.ulaval.ca (A.W.); julien.boucher@crchudequebec.ulaval.ca (J.B.); henintsoa.rabezanaha@crchudequebec.ulaval.ca (H.R.); 2 Département de Microbiologie-Infectiologie et d’Immunologie, Faculté de Médecine, Université Laval, Quebec City, QC G1V 0A6, Canada

**Keywords:** influenza viruses, extracellular vesicles, infectious virus particles, light scattering, dot blot

## Abstract

Influenza virus is one of the most frequent causes of respiratory infection in humans. Recent studies suggest that extracellular vesicles (EVs)—small particles released by cells during influenza virus infection—can influence the immune response and viral pathogenesis. However, during viral replication, infected cells can also release EVs, which may include different subtypes. This study aimed to purify and characterize viral preparations and EVs using sequential ultracentrifugation methods. Influenza A/H1N1, A/H3N2, and B virus strains were produced in human Calu-3 and A549 cell lines. Viral supernatants then underwent a series of differential ultracentrifugation steps at 3000× *g*, 17,000× *g*, and 100,000× *g*. Dynamic light scattering analysis (DLS) validated size heterogeneity for all three types of EVs. Measurement of infectious virus particles for all three pellets showed virus enrichment at 17,000× *g* and 100,000× *g*. Dot blot analysis confirmed the enrichment of virus particles in these fractions and the presence of EV protein. This study demonstrates the presence of EVs in virus preparations and highlights the need for improved separation methods to characterize them better and explore their role in viral infection pathogenesis.

## 1. Introduction

Influenza epidemics cause a significant global health burden, with approximately one billion cases annually, including 3 to 5 million severe cases, and 250,000 to 650,000 deaths [1]. Influenza viruses (IVs), belonging to the *Orthomyxoviridae* family, are divided into four types: A, B, C, and D. Influenza A and B viruses circulate in humans and are the leading causes of seasonal epidemics. In addition, influenza A viruses have caused several pandemics over the last 100 years [2,3,4]. Influenza A viruses are divided into subtypes such as A/H1N1, A/H3N2 and A/H5N1, based on their distinguishable surface glycoproteins -hemagglutinin (HA) and neuraminidase (NA), which can reassort due to antigenic shifts [5]. The annual evaluation of influenza vaccine composition is necessary due to the virus’s constant evolution through antigenic changes [6,7]. Studying seasonal strains and understanding influenza virus pathogenesis is crucial for developing prevention and treatment strategies [8]. Recent studies indicated that influenza virus-infected cells release extracellular vesicles (EVs), and these investigations are beginning to elucidate their functional roles during viral infection [9,10,11].

EVs are nanoparticles released by cells of prokaryotic and eukaryotic organisms, bound by a lipid bilayer, and incapable of self-replicating [12,13]. They can be secreted by various cell types, including epithelial, endothelial, immune, and blood cells [12], and purified from body fluids (plasma, urine, saliva, breast milk, semen, cerebrospinal fluid) and cell culture supernatants [14]. EVs can be classified into exosomes, microvesicles, and apoptotic bodies based on their biogenesis, cellular source, release pathways, size, content, and function [15,16]. Exosomes, approximately 30 to 150 nm, are intraluminal vesicles within multivesicular bodies released to the extracellular environment upon their fusion with the plasma membrane, while microvesicles (100 to 1000 nm) are derived from the budding of the plasma membrane. Apoptotic bodies are larger vesicles ranging between 1 and 4 µm, and are released from apoptotic cells [15,16,17,18].

In physiological conditions, signaling between cells is important for maintaining the equilibrium and homeostasis of all multicellular organisms, and numerous studies demonstrate that EVs are a key factor in intercellular communication [10,11,19,20]. Indeed, EVs contain nucleic acids (DNA, mRNA, and microRNA), several proteins, and lipids, and they can transfer their cargo between cells, participating in intercellular communication and transformation [10]. A variety of proteins associated with EVs, especially with exosomes, are involved in membrane fusion (flotillin, GTPases, annexins, Rab) and are found in endosomal sorting complex required for transport (ESCRT) like tetraspanins (CD9, CD63, CD81), heat shock proteins (HSPs), ALG-2-interacting protein X (ALIX) and tumor susceptibility gene 101 (TSG101) [21,22]. EVs have distinct protein profiles due to their various formation pathways; some proteins serve as biomarkers for their isolation or detection [23].

A range of methodologies, including ultracentrifugation, size-based techniques, and Immunoaffinity Capture-Based, are used to separate and concentrate EVs [13,16,18,24]. The majority of studies use isolation by differential ultracentrifugation techniques, whereby centrifugal force separates vesicles based on their size and density. Generally, centrifugal force at 300 to 3000× *g* is applied to separate larger particles, at 10,000× *g* to 17,000× *g* to remove vesicles bigger than 100 nm, and finally at 100,000× *g* to pellet exosomes [14,25,26]. Once EVs are purified, they are characterized and quantified based on their size, concentration, and purity [16], using different methods such as flow cytometry, nanoparticle tracking analysis, multi-angle light scattering, dynamic light scattering (DLS), electron microscopy, quantitative PCR, and Western blot [13,18,22].

Recent studies highlight the role of EVs in viral pathogenesis, including their involvement in transmission, immune modulation, and evidence that exosomes contribute to the spread of HIV, HCV, Dengue, and Human T-cell lymphotropic virus infections [27,28]. Another study revealed that small bioengineered EVs can deliver several antigenic particles of severe acute respiratory syndrome coronavirus 2 and induce a broad immune response, activating T and B-lymphocytes [29].

In the case of influenza virus, several reviews have explored the role of EVs in influenza virus infections [9,10,11,30,31,32]. Bedford et al. showed that exosomes secreted into the respiratory tract during infection triggered pulmonary inflammation and carried viral antigens for immune activation [9]. Zabrodskaya et al. reported that exosomes from influenza A virus-infected cells contained regulatory miRNAs and mRNAs that enhanced viral replication in neighboring cells while suppressing inflammatory gene expression in A549 cells [10]. Similarly, Ge et al. revealed that exosomes secreted by H1N1pdm09-infected Madin-Darby canine kidney (MDCK) and A549 cells supported viral RNA production and revealed infection-induced changes in miRNAs affecting interferon, apoptosis, and MAPK pathways [32]. Thus, EVs play multiple roles in the pathogenesis of influenza infection.

However, most of these studies have focused on influenza A virus-derived EVs, while those released during influenza B infection remain poorly characterized. Although influenza A viruses (IAV) have been extensively studied, influenza B viruses (IBV) remain comparatively underexplored, particularly regarding their interaction with host cells and the release of extracellular vesicles (EVs). Most existing studies on virus-derived EVs have focused on IAV, while EVs produced during IBV infection are still poorly characterized. This imbalance has left a significant knowledge gap, even though influenza B contributes substantially to the global seasonal influenza burden and exhibits distinct patterns of circulation, host restriction, and immune response.

A comparative analysis of EVs released during influenza A and B infections is therefore essential to better understand virus-specific EV signatures and their potential implications for viral pathogenesis, immune modulation, and transmission. Antigenically, influenza A and B viruses belong to distinct genera and display substantial genetic and structural differences in their surface glycoproteins, particularly HA and NA [6]. Despite these differences, both virus types co-circulate during annual epidemics and are jointly included in trivalent or quadrivalent seasonal influenza vaccines to ensure broad-spectrum protection [7]. This highlights the biological and epidemiological relevance of comparing EV populations derived from both virus types within physiologically relevant human models.

Moreover, in this study, we selected two human respiratory epithelial cell lines, A549 and Calu-3, that together represent complementary regions of the airway epithelium. A549 cells, derived from human alveolar type II epithelial cells, are widely used in influenza research because of their high susceptibility to viral replication and their relevance to the lower respiratory tract [33]. Calu-3 cells, originating from human bronchial epithelium, are recognized as a well-established model for studying respiratory virus infection and epithelial barrier responses [34]. While A549 cells have been frequently employed to investigate EV release during influenza A infection, to our knowledge, studies describing EVs produced by influenza-infected Calu-3 cells remain extremely limited or nonexistent. Using both A549 and Calu-3 cell lines provides complementary perspectives on EV production by influenza-infected human airway epithelial cells, reflecting distinct epithelial origins within the respiratory tract.

Overall, although studies highlight the roles of EVs, especially exosomes, during influenza infection, few have considered the different subtypes of EVs produced and evaluated a comparison of the molecular profiles of EV products generated by distinct subtypes of influenza A and influenza type B viruses, as has been observed with HIV [25]. The characteristics of EV subtypes related to influenza A and B infection are still largely unknown. Therefore, further investigation of EVs in the context of influenza infection is necessary to understand their contribution. This study aimed to purify, characterize, and compare EVs produced in A549 and Calu-3 cell lines infected with seasonal A/H1N1, A/H3N2, and B influenza viruses included in the trivalent influenza vaccine for the 2023–2024 season.

## 2. Materials and Methods

### 2.1. Cell Lines

Human lung epithelial Calu-3 cells (HTB-55 ™) and human pulmonary epithelial A549 cells (CCL-185 ™) were obtained from American Type Culture Collection (ATCC^®^) and cultured in Dulbecco’s Modified Eagle’s Medium (DMEM, Gibco, New York, NY, USA) complemented with 10% Fetal Bovine Serum (FBS, HyClone Laboratories, Logan, UT, USA). The Calu-3 cells’ medium was supplemented with 2% MEM non-essential amino acid solution 100× (Gibco), 0.05% sodium bicarbonate (Gibco), 5000 U/mL penicillin-streptomycin solution (Gibco), and 1 mM sodium pyruvate (Gibco). Cultures were maintained at 37 °C in a humidified incubator with 5% CO_2_.

MDCK over-expressing the α2,6 (MDCK α2,6) sialic acid receptor cells were kindly provided by Y. Kawaoka from the University of Wisconsin, Madison, WI [35] and grown at 37 °C with 5% CO_2_ in Eagle’s Minimum Essential Medium (EMEM, Gibco) supplemented with 10% FBS, 1% HEPES, and 0.1% puromycin (7.5 μg/mL of puromycin, Wisent Inc, Saint-Jean-Baptiste, QC, Canada).

### 2.2. Viruses

Three influenza viruses recommended by the WHO for the 2023–2024 seasonal trivalent influenza vaccine: A/Victoria/4897/2022 (H1N1) pdm09-like virus (A/Vic), A/Darwin/9/2021 (H3N2)-like virus (A/Dar), and B/Austria/1359417/2021 (B/Victoria lineage)-like virus (B/Aus) were used in this study. They were purchased from the National Institute for Biological Standards and Control (NIBSC, Potters Bar, Hertfordshire, UK).

### 2.3. Stock Viral Production

Confluent Calu-3 and A549 cells were washed twice with phosphate-buffered saline (PBS) prior to infection to remove FBS. Cells were infected with viruses at different multiplicities of infection (MOI) (Table 1) as previously described [32]. Viruses were diluted in EMEM without serum supplemented with HEPES and 1 μg/mL of tosyl phenylalanyl chloromethyl ketone (TPCK)-trypsin (Thermo Fisher Scientific, Waltham, MA, USA), then added to cells. Infected cultures were incubated at 37 °C for virus type A and 34 °C for virus type B until ~90% cytopathic effect (CPE) was observed. Uninfected control cells were treated under the same conditions.

### 2.4. Virus Titration

Viral titers were determined using two complementary methods: the 50% tissue culture infectious dose (TCID_50_) and the plaque-forming unit (PFU) assays [36]. For the TCID_50_ assay, MDCK α2,6 cells were seeded in 96-well plates (5 × 10^3^ cells/100 µL) and cultured for 3 days at 37 °C with 5% CO_2_ until 80–90% confluency. After PBS washing, 180 µL of MEM supplemented with TPCK (1 µg/mL) was added to each well, followed by a ten-fold serial dilution of the viral stock in quadruplicate. CPE was recorded after 4 days, and the infectious titer was calculated using the Reed and Muench method [37]. In parallel, the PFU assay was performed by seeding MDCK cells (1 × 10^5^ cells/2 mL) in 6-well plates. After 3 days, monolayers were washed, and 500 µL of virus diluted from 10^−2^ to 10^−7^ were added in triplicate. Following a 1-h adsorption period, cells were overlaid with infection media containing 1.6% agarose and 2× EMEM with TPCK-trypsin. After 3 days of incubation, cells were fixed with 4% formaldehyde and stained with crystal violet. Viral titers were expressed in PFU/mL based on the average plaque count and dilution factor.

### 2.5. Purification of EVs by Ultracentrifugation

**Figure 1:**
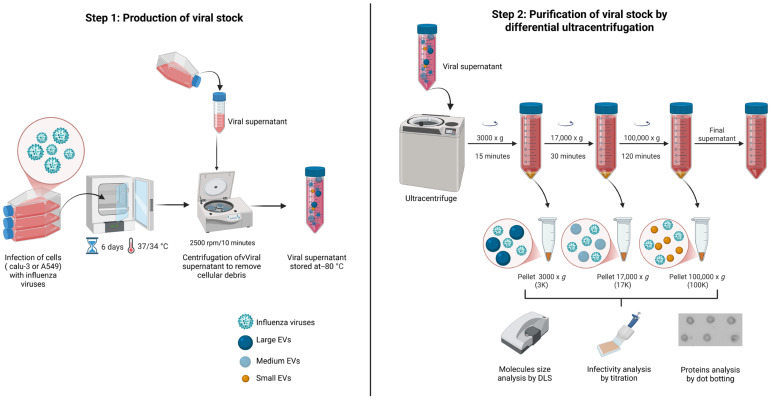
Schematic representation of the two-step process used for viral stock preparation and EV co-purification. Step 1: Calu-3 or A549 cells were infected with influenza viruses, and the viral supernatants were collected upon reaching an almost total cytopathic effect, approximately 6 days post–infection. Supernatants were clarified by low-speed centrifugation and stored at −80 °C. Step 2: The clarified supernatants were subjected to sequential centrifugations at 3000× *g*, 17,000× *g*, and 100,000× *g* to obtain the 3K, 17K, and 100K pellets. Each pellet was resuspended in PBS and is expected to be enriched in viral particles and large, medium, or small EVs, respectively. The resulting fractions were analyzed for particle size by DLS, infectivity by titration, and protein content by dot blotting. Created in https://BioRender.com (accessed on 7 October 2025).

Collected supernatants from viral stock production were used to co-purify EVs as previously described [26]. Three successive centrifugations at 3000× *g* (15 min at 4 °C), 17,000× *g* (30 min at 4 °C), and 100,000× *g* (120 min at 4 °C) using a rotor T1250 (Thermo Fisher Scientific, Waltham, MA, USA) for the ultracentrifuge Sorvall WX Ultra Series (Thermo Fisher Scientific, Waltham, MA, USA) were executed. All pellets were resuspended in 0.20 µm-filtered PBS 1X in 1/20th of the original volume, and EVs were promptly stored at −80 °C until further analysis. EVs from the medium of mock-infected cells were monitored in the same way. Fractions obtained after centrifugation are named as 3K for 3000× *g*, 17K for 17,000× *g* and 100K for 100,000× *g (*Figure 1).

### 2.6. Dynamic Light Scattering Analysis

EVs’ hydrodynamic size was determined using dynamic light scattering (DLS) with a Zetasizer NanoS (Malvern Instruments Ltd., Malvern, UK) as described before [26]. The purified EVs were diluted to 1/10 using sterile PBS; then, 100 μL were transferred into a cuvette, and intensity correlation functions of particle samples were measured. For each sample, the values presented in this study are the average of two measurements per sample, taken at a temperature of 25 °C, with a detection angle of 173° and a laser beam wavelength of 633 nm. Results were assessed using the Malvern Zetasizer software 8.01.4906, based on the CONTIN analysis algorithm, and homogeneity or size of particles was recognized through the percent intensity or radius, respectively [38]. The measurements also provided the sample-derived count rate. This parameter, expressed in kilo counts per second (kcps), corresponds to the number of photons scattered by nanoparticles per second.

### 2.7. Antibodies Analyzed

EV-associated proteins were detected using specific antibodies. For that, anti-CD9 mouse monoclonal antibody (Cat: CBL162, MilliporeSigma, Burlington, MA, USA), anti-flotillin 2 Polyclonal Antibody (Cat: PA5-79268, Thermo Fischer Scientific), and anti-HPS70/HSC70 (W27) mouse monoclonal antibody (sc-24, Santa Cruz Biotechnology, Santa Cruz, CA, USA) were used.

Pan Influenza A Nucleoprotein Antibody Mouse Mab Cat: 40205-MM16, Pan Influenza B Nucleoprotein Antibody, Mouse Mab Cat: 40438-MM10, Influenza A H3N2 Hemagglutinin/HA Antibody, Mouse Mab Cat: 11056-MM03, Influenza B Hemagglutinin/HA Antibody, Rabbit Mab Cat: 11053-R004, all purchased from SinoBiological (Chesterbrook, PA, USA) were used to detect influenza virus proteins. A Goat anti-mouse IgG (H+L) secondary antibody (Cat: 31430, Thermo Fisher Scientific, Waltham, MA, USA) and a Goat Anti-Rabbit IgG (H+L) secondary antibody (Cat: 111-035-003, Jackson ImmunoResearch Laboratories Inc., West Grove, PA, USA), both coupled to horseradish peroxidase, were used.

### 2.8. Dot Blotting Assay

The qualifications of the protein contained in the starting material (cell culture supernatant) samples and EV preparations were shown by the immunoblot method described previously [10]. Two µL of samples were applied to a dried nitrocellulose membrane (Bio-Rad Laboratories, Hercules, CA, USA), which was first saturated in distilled water, followed by PBS washing. After drying, the membrane was incubated with a 5% blocking solution containing Blotting Grade Blocker Non-Fat Dry Milk (Bio-Rad Laboratories) in 0.1% PBST (PBS-Tween-20) for 1 h. The blotting reagent was removed, the membrane was washed with PBST for 5 min, and each primary antibody diluted in 2% blocking solution to 1 µg/mL was added and incubated for 1 h with gentle rotation at room temperature (RT). The membrane was washed with PBST twice and incubated with a 1:1000 secondary antibody solution for 1 h at RT. The membrane was washed twice with PBST (10 min), and spots were visualized using the Clarity Western ECL Substrate kit (Bio-Rad Laboratories) and a ChemiDoc™ imaging station (Bio-Rad Laboratories).

### 2.9. Statistical Analysis

All statistical analyses were performed using GraphPad Prism version 9.5.1. Results are presented as the mean of at least three independent experiments, unless otherwise specified. Statistical tests, including two-way ANOVA, were applied and are indicated in the corresponding figure caption. *p*-values less than 0.05 were considered statistically significant and were shown in the graphic. Statistical significance is represented as follows: * for *p* < 0.05, ** for *p* < 0.01, *** for *p* < 0.001, and **** for *p* < 0.0001.

## 3. Results

### 3.1. Viral Load Comparison of Influenza Strains

**Figure 2:**
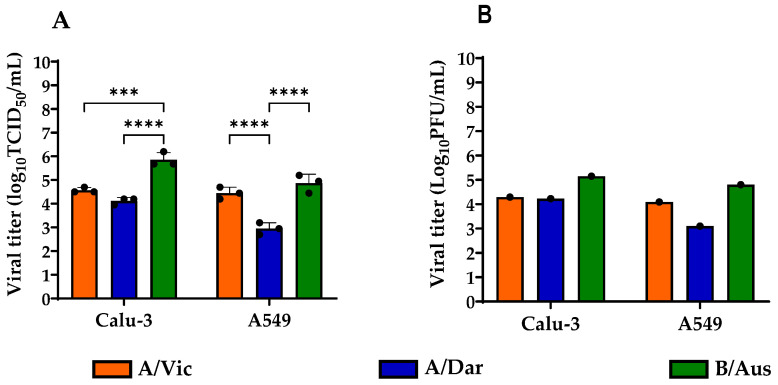
Viral Titers of Influenza Virus Stocks. A/Vic, A/Dar, and B/Aus viruses were produced in Calu-3 and A549 cells and titrated using both TCID_50_ and PFU assays. (**A**) Viral titers of the three strains were determined by the TCID_50_ assay. Data represent the mean of three replicates from three independent experiments. (**B**) Viral titers determined by the PFU assay. Data represents the mean of three technical replicates from a single experiment. Graphs were generated using GraphPad Prism version 9.5.1. Statistical analysis was performed using two-way ANOVA. **** *p* < 0.0001; *** *p* < 0.001.

Three seasonal strains recommended by the WHO for the 2023–2024 vaccine, A/Vic, A/Dar, and B/Aus, were used in this study. In vitro production of these viruses yielded viral stocks, which were titrated using two distinct methods: TCID_50_ and PFU assays. The results demonstrate that these viruses are replicated efficiently at specific MOIs, depending on the virus in each cell line, to enhance detection of viral replication (Table 1). Titration results of the three seasonal strains (A/Vic, A/Dar, and B/Aus) produced in Calu-3 and A549 cells showed higher TCID_50_/mL titers for all three viruses in Calu-3 compared to A549 cells (Figure 2A). The average viral titers in Calu-3 cells were 4.57 log_10_ TCID_50_/mL for A/Vic, 4.2 log_10_ TCID_50_/mL for A/Dar, and 5.85 log_10_ TCID_50_/mL for B/Aus. In contrast, the corresponding titers in A549 cells were 4.45, 2.95, and 4.87 log_10_ TCID_50_/mL, respectively. Analysis of the data revealed a significant difference in the replication of the different strains within the same cell line only by the TCID_50_ assay (Figure 2A). The B/Aus strain exhibited significantly higher titers compared to A/Vic and A/Dar in Calu-3, and to A/Dar in A549 cells. Similar trends were observed using the plaque assay, although the differences in titers were not statistically significant (Figure 2B). The average titers of A/Vic, A/Dar, and B/Aus in Calu-3 cells were 4.29, 4.23, and 5.13 log_10_ PFU/mL, respectively, and 4.09, 3.10, and 4.80 log_10_ PFU/mL in A549 cells. In summary, Calu-3 and A549 cells showed suitable in vitro production of influenza A and B viruses in our laboratory setting and were used for subsequent studies.

### 3.2. Analysis of Size Distribution and Heterogeneity of Co-Purified EVs and Viruses

Supernatants from virus-infected cell cultures were subjected to sequential centrifugation to obtain three pellet fractions, 3K, 17K, and 100K, which. Then, the hydrodynamic size of EVs and viruses in each pellet was determined by using the Zetasizer Nano system.

This instrument operates on the principle of DLS to assess the Brownian motion of nanoparticles in suspension, allowing for the characterization of hydrodynamic size, sample heterogeneity, and relative particle abundance. Control samples were obtained from uninfected cell culture supernatants processed under the same conditions.

**Figure 3:**
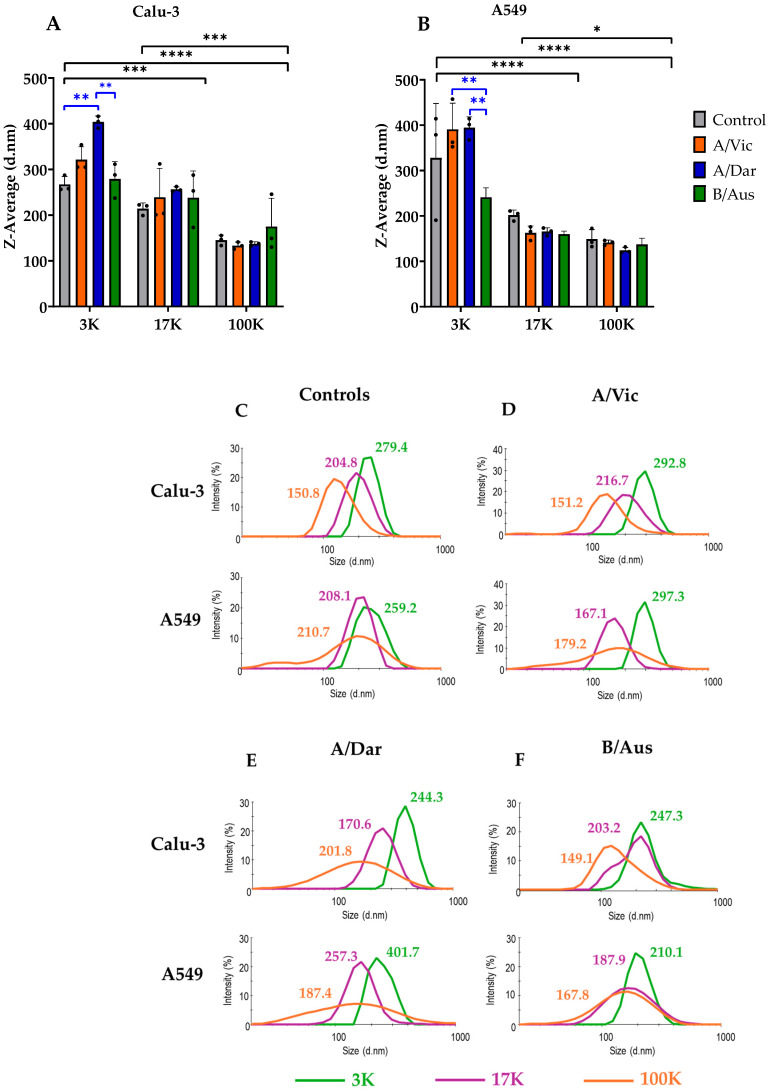
Size and Heterogeneity of EVs and Co-Purified Viruses from Viral Productions. The graphs in this figure are based on data obtained from dynamic light scattering (DLS) analysis. (**A**) Z-average values of nanoparticles detected in each pellet fraction from control and virus-infected Calu-3 cell supernatants. (**B**) Z-average values of nanoparticles from control and virus-infected A549 cell supernatants. (**C**) Size distribution profiles of nanoparticles from control samples, plotted as a function of intensity. (**D**–**F**) Size distribution profiles of nanoparticles from virus-infected samples with A/Vic (**D**), A/Dar (**E**), and B/Aus (**F**), respectively. Data shown in graphs A and B represent the mean of three biological replicates. Graphs (**C**–**F**) display averaged intensity profiles generated by the Malvern Zetasizer software 8.01.4906 from three biological replicates. For each measurement, two technical replicates were analyzed. Graphs A and B were generated using GraphPad Prism version 9.5.1. Statistical analyses were performed using two-way ANOVA. Black asterisks indicate significant differences between the 3K, 17K, and 100K fractions pooled across all cell lines. Blue asterisks indicate significant differences in nanoparticle sizes between fractions within each cell line. **** *p* < 0.0001; *** *p* < 0.001; ** *p* < 0.01; * *p* < 0.05.

DLS analysis was performed on the 3K, 17K, and 100K fractions derived from both control cell culture supernatants and virus production in Calu-3 (Figure 3A) and A549 (Figure 3B) cells. For each pellet, the Z-average was obtained, representing the intensity-weighted mean hydrodynamic diameter of the nanoparticle population. Size distribution profiles revealed a consistent trend across all samples: particles in the 3K fractions were the largest, those in the 17K fractions were intermediate, and those in the 100K fractions were the smallest. No significant differences were observed in the size of nanoparticles among uninfected control samples from the two cell lines. However, analysis of the fractions from A/Vic, A/Dar, and B/Aus virus productions in Calu-3 and A549 cells revealed subtle but significant differences in nanoparticle size for both cell lines (Figure 3A,B). These differences in size distribution were further supported by representative intensity-based distribution curves (Figure 3C–F), where the main peaks corresponded to size ranges consistent with typical EVs. In general, the 3K fractions contained the largest nanoparticles (100–1000 nm), the 17K fractions contained intermediate-sized particles (100–500 nm), and the 100K fractions included the smallest particles. Specifically, in control conditions, the largest EVs in the 3K fractions measured 279.4 nm (Calu-3), and 259.2 nm (A549); the 17K fractions contained slightly smaller EVs (204.8 nm and 208.1 nm, respectively); and the 100K fractions harbored the smallest vesicles (150.8 nm and 210.7 nm) (Figure 3C). This pattern was observed in both Calu-3 (Figure 3A) and A549 cells (Figure 3B) for each virus. Nevertheless, significant differences in particle sizes were detected among the 3K fractions across the three viral strains and both cell lines.

Furthermore, Figure 3C–F also illustrate the heterogeneity of nanoparticles present in each fraction. Heterogeneity was evaluated based on the number and width of peaks in the intensity distribution curves. A higher number of peaks and broader peak widths are indicative of greater heterogeneity, while a single narrow peak suggests a more homogeneous population. This analysis revealed that the EV populations studied in this project exhibit varying degrees of heterogeneity across the three pellet fractions. In particular, the 3K fractions represented narrow peaks, suggesting relatively homogeneous populations; the 17K fractions showed broader peaks; and the 100K fractions exhibited the widest peaks, indicating a higher degree of size variability within the population. Thus, the distribution curves indicated particle size heterogeneity across all viral preparations, suggesting the presence of three EV subtypes in each sample. This heterogeneity was further supported by the polydispersity index (PDI) values shown in Table 2. According to the manufacturer’s guidelines (Malvern Panalytical), a PDI between 0.0 and 0.1 indicates a highly monodisperse (homogeneous) sample, 0.1 to 0.4 indicates moderate polydispersity, and values above 0.4 reflect high polydispersity (heterogeneity). Taken together, the data confirmed that all three EV subtypes are composed of heterogeneous nanoparticle populations, with no clear influence from the virus strain or cell type on the degree of heterogeneity. These findings indicate that the applied purification method enables the detection of EVs and viral particles co-purified in samples, as reflected by size distribution profiles.

### 3.3. Relative Quantification of Nanoparticles in EV and Virus Pellets

**Figure 4:**
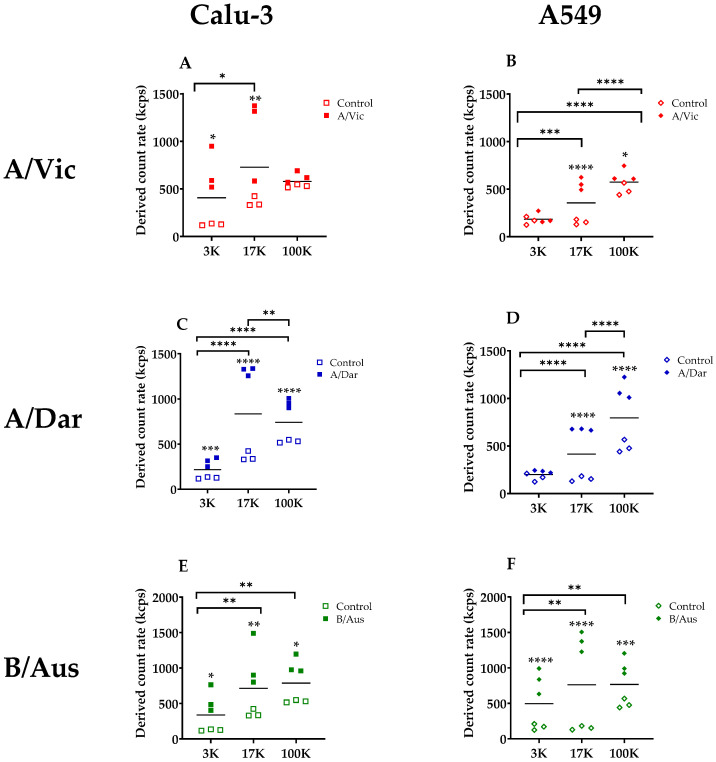
Relative Abundance of Nanoparticles in Purified Pellet Fractions. Relative quantification of nanoparticles in the 3K, 17K, and 100K pellet fractions obtained from control and virus-infected cell cultures, as measured by dynamic light scattering (DLS). Square symbols indicate Calu-3 samples, while diamond symbols indicate A549 samples. Virus productions are color-coded: A/Vic in red, A/Dar in blue, and B/Aus in green. (**A**,**B**) represents the relative nanoparticle abundance in A/Vic virus stock compared to EV controls, (**C**,**D**) represents the relative nanoparticle abundance in A/Dar virus stock, and (**E**,**F**) shows the relative nanoparticle abundance in B/Aus virus stock compared to EV controls. Each data point represents the mean of three technical replicates, and each condition was assessed using three independent biological replicates. Statistical comparisons were performed using Two-way ANOVA with GraphPad Prism version 9.5.1. Two levels of significance testing are indicated: asterisks positioned on data points denote differences between infected and control samples within the same fraction, while horizontal bars indicate differences between pellet fractions (3K, 17K, and 100K), regardless of infection status. **** *p* < 0.0001; *** *p* < 0.001; ** *p* < 0.01; * *p* < 0.05.

DLS analysis using the Zetasizer also provided a relative quantification of nanoparticles present in each pellet fraction for both viral and control EV preparations (Figure 4). The relative abundance of nanoparticles in the 3K, 17K, and 100K fractions was compared between virus-infected samples and uninfected controls. In Calu-3 cells (Figure 4A,C,E), all three viruses induced a significant increase in nanoparticle production in at least two pellet fractions. For A/Vic, significant increases were observed in the 3K (*p* < 0.05) and 17K (*p* < 0.01) fractions, with no significant difference in the 100K fraction. A/Dar infection resulted in a highly significant increase across all three fractions (*p* < 0.001 to *p* < 0.0001), particularly in the 17K fraction. Similarly, B/Aus infection led to a significant rise in nanoparticle abundance in all fractions (*p* < 0.05 to *p* < 0.01). In A549 cells, similar trends in nanoparticle abundance were detected (Figure 4B,D,F). For the A/Vic strain, relative nanoparticle abundance was significantly increased in the 17K (*p* < 0.0001) and 100K (*p* < 0.05) fractions. A/Dar infection led to significant increases in the 17K and 100K fractions as well (*p* < 0.001). Likewise, B/Aus infection resulted in elevated nanoparticle levels across all fractions (*p* < 0.01 to *p* < 0.0001), with particularly high values in the 17K and 100K fractions. Overall, the 17K fraction appeared to be the most enriched in nanoparticles, especially in Calu-3 cells, whereas the 100K fraction was more enriched in A549 cells. This trend was consistently observed for both influenza A strains. In contrast, for the influenza B virus, no significant difference in nanoparticle abundance was observed between the 17K and 100K fractions in either cell line. Overall, viral infection led to a significant increase in the relative abundance of nanoparticles in both cell types, although the extent of this increase varied depending on the pellet fraction and cell line. Comparative analysis of infected and control conditions revealed that, for each fraction, nanoparticle levels were significantly higher in virus-infected samples than in EV controls. This increase in nanoparticle abundance was observed across all three viral strains (A/Vic, A/Dar, and B/Aus), although the magnitude of the effect varied by virus, cell type, and pellet.

These differences in relative abundance raised the hypothesis that specific fractions might be more enriched in infectious particles, prompting an infectivity assessment (see the following section).

### 3.4. Assessment of Infectivity in EV-Associated Fractions

**Figure 5:**
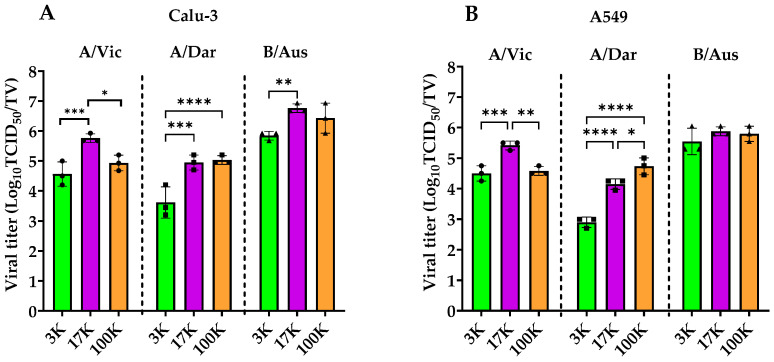
Infectivity of Influenza Particles Associated with Purified EV-Containing Fractions. Viral titers in the 3K (green), 17K (purple), and 100K (orange) pellet fractions were determined using TCID_50_. (**A**,**B**) represent TCID_50_ titers per total volume (TV) for A/Vic, A/Dar, and B/Aus viruses produced in (**A**) Calu-3 cells and (**B**) A549 cells. For each virus, TCID_50_ values represent the mean of three technical replicates from three independent biological samples. Graphs were generated using GraphPad Prism version 9.5.1. Statistical analysis was performed using two-way ANOVA. **** *p* < 0.0001; *** *p* < 0.001; ** *p* < 0.01; * *p* < 0.05.

To evaluate the infectivity of viruses present in the different pellet fractions, viral titers were measured using both TCID_50_ and PFU assays. Infectious virus particle quantifications by TCID_50_ for the three fractions derived from the production of A/Vic, A/Dar, and B/Aus are shown in Figure 5A,B, while titers obtained using the PFU assay are shown in Supplementary Figure S1. The results indicate that the distribution of infectious particles varies depending on the virus strain and the cell line, although consistent trends were observed. In Calu-3 cells (Figure 5A), viruses exhibited significantly lower titers in the 3K fraction compared to the 17K (A/Vic and A/Dar) and 100K (A/Dar) fractions (*p* < 0.05 to *p* < 0.0001). B/Aus followed a similar pattern, with titers comparable to those of A/Vic and A/Dar. In A549 cells (Figure 5B), most infectious particles were concentrated in the 17K and 100K fractions, particularly for A/Vic and A/Dar. Interestingly, PFU-based titration revealed no significant differences in viral titers between the three fractions in either cell line, in contrast to the TCID_50_ results (see Supplementary Figure S1). Overall, enrichment of infectious particles in the 17K and 100K fractions was more clearly detected using the TCID_50_ assay. This trend was especially pronounced for the A/Vic and A/Dar strains, whereas B/Aus appeared to have a more uniform distribution of infectious particles across all three fractions. The 17K fraction was identified as a key site for the recovery of infectious particles, which are co-purified with EVs in both the 17K and 100K fractions. These findings suggest potential differences in replication and release mechanisms between influenza A and B virus strains.

### 3.5. Characterization of Co-Purified EVs and Viral Particles by Immunoblotting

**Figure 6:**
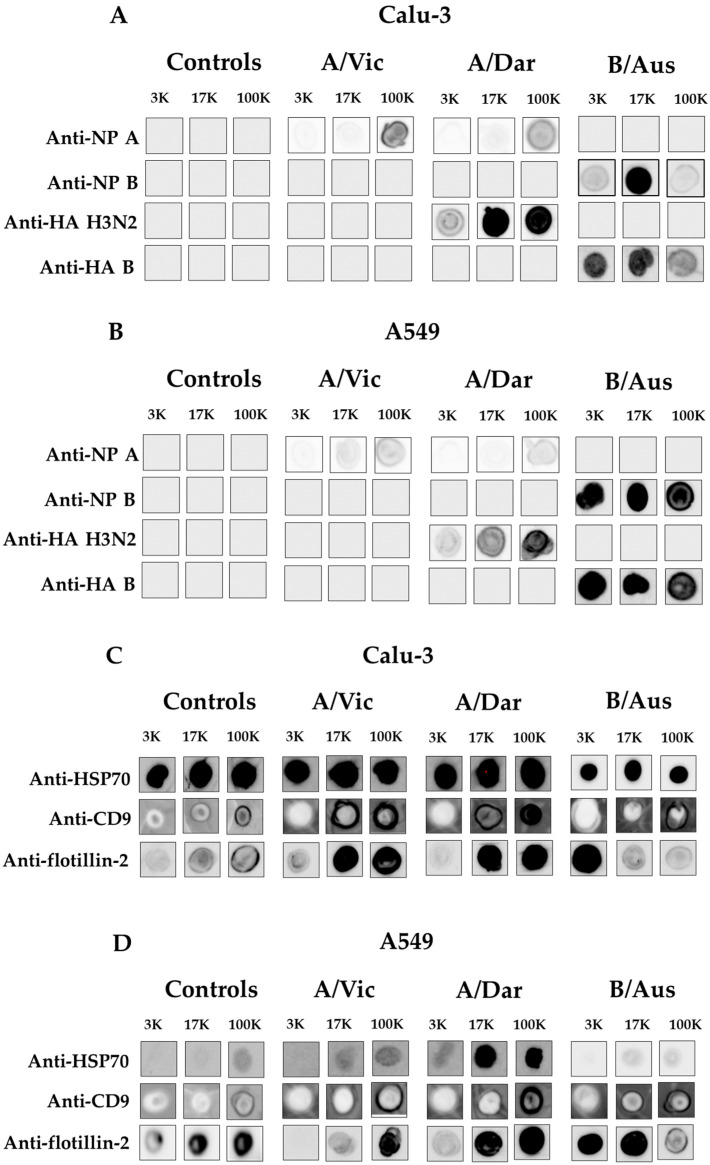
Protein Characterization of Viral Particles and EVs in the 3K, 17K, and 100K pellets. Immunoblotting was performed using anti-NP and anti-HA antibodies to detect influenza viral proteins in the fractions, and antibodies against HSP70 (a heat shock protein), CD9 (transmembrane protein of the tetraspanin family), and flotillin-2 (membrane-associated protein linked to lipid rafts) to detect EV-associated proteins. Detection of viral (**A**) and EV (**C**) proteins in fractions derived from A/Vic, A/Dar, and B/Aus production in Calu-3 cells. Detection of viral (**B**) and EV (**D**) proteins in fractions derived from A/Vic, A/Dar, and B/Aus production in A549 cells. Uninfected controls were included for each condition. Sample processing controls are run on different membranes.

To confirm the presence of viral particles in the 3K, 17K, and 100K fractions and to identify the pellets in which they were enriched, immunoblot analysis was performed. Antibodies targeting conserved viral proteins, such as the nucleoprotein (NP) of influenza A and B viruses, as well as strain-specific HA of H3N2 and influenza B, were used to characterize the viral components present in each pellet. In parallel, the presence of EVs was assessed using antibodies against established EV markers: HSP70 (a cytosolic chaperone), CD9 (an exosomal surface marker), and flotillin-2 (a microvesicle marker). The relative intensity of the detected signals was used as a qualitative or semi-quantitative indicator to estimate the distribution of viral proteins and EV markers across different fractions. Thus, immunoblot analysis revealed the presence of viral NP and HA proteins in the 3K, 17K, and 100K fractions following infection with A/Vic, A/Dar, and B/Aus in Calu-3 (Figure 6A) and A549 cells (Figure 6B). Uninfected cell controls were included for each condition. Antibodies against influenza A NP and H3N2 HA allowed the specific detection of A/Vic and A/Dar, while influenza B NP and HA antibodies specifically detected B/Aus. As expected, no NP or HA signals were detected in any fraction from uninfected samples in either cell line, confirming the absence of viral contamination. In infected samples, viral proteins were predominantly detected in the 17K and 100K fractions, although signal intensities varied depending on the virus and cell line. In Calu-3-derived fractions, NP was detected across all three fractions for A/Vic and A/Dar, with the strongest signal observed in the 100K pellet. H3N2 HA detection followed a similar pattern, with A/Dar HA signals enriched in the 17K and 100K fractions. For B/Aus, NP was mainly enriched in the 17K and 100K fractions, while HA was more abundant in the 3K and 17K fractions. A similar distribution was observed in A549 cells. Influenza A NP and H3N2 HA from A/Vic and A/Dar were present in all three fractions, again with higher intensity in the 100K pellet. For B/Aus, both NP and HA were detected in the 3K, 17K, and 100K fractions with comparable signal intensities. These results indicate that viral proteins from influenza A viruses are primarily enriched in the 100K fraction, with detectable levels also present in the 17K fraction. In contrast, influenza B viral proteins appear to be more evenly distributed across all three fractions.

Regarding the enrichment profiles of the EV-associated proteins HSP70, CD9, and flotillin-2 in fractions derived from virus-infected Calu-3 cells (Figure 6C), HSP70 was found to be all three fractions (3K, 17K, and 100K) for each of the three viruses. CD9 was enriched in both 17K and 100K fractions, with higher signal intensity observed in the 100K fraction. Flotillin-2 was primarily detected in the 17K and 100K fractions for influenza A viruses, and in the 3K fraction for the influenza B virus. Overall, EV marker signals were stronger in infected samples compared to uninfected controls. In A549 cells (Figure 6D), EV markers were also enriched mainly in the 17K and 100K fractions for A/Vic and A/Dar, although signal intensities were generally lower than those observed in Calu-3 cells. In contrast, for B/Aus, HSP70 and CD9 were enriched in the 17K and 100K fractions, while flotillin-2 was more abundant in the 3K and 17K fractions. Altogether, these findings highlight notable differences in the release mechanisms or vesicle-associated particle organization between influenza A and B viruses.

## 4. Discussion

Influenza A and B viruses are major respiratory pathogens with a high public health impact. Studying both pandemic and recently circulating seasonal strains is essential for improving vaccine effectiveness, given their continuous antigenic drift and reassortment [6,39,40,41]. EVs, including exosomes, microvesicles, and apoptotic bodies, are increasingly recognized as contributors to influenza pathogenesis. They were carrying viral components, modulating immune responses [10,42], and altering host pathways [9,14,22,43,44,45]. For instance, IAV-infected human epithelial A549 cells produce exosomes able to inhibit viral replication by inducing interferon production [46]. While the diversity of EVs, only exosomes are the most studied in the influenza context; however, investigating other subtypes could provide a more comprehensive view of virus–host interactions. Moreover, data on EV compositional changes during infection are almost nonexistent for influenza B virus. This study was therefore based on the hypothesis that viral infection generates distinct EV subpopulations differing in size and composition, and that this diversity varies with the infected cell type. Based on this hypothesis, the objectives were to produce the three viral strains included in the WHO-recommended 2023–2024 Northern Hemisphere influenza vaccine in human Calu-3 and A549 cell lines.

Our results showed that both Calu-3 and A549 cell lines supported the production of the influenza strains studied, with MOIs adjusted according to each cell line’s permissiveness, as determined by our preliminary assays (see Supplementary Table S1). Calu-3 cells were generally more permissive than A549 cells, producing higher titers for all viruses tested from the same inoculum (5 log_10_TCID_50_), with B/Aus showing the highest titers, followed by A/Vic and A/Dar. Final MOIs yielded replication in all conditions, though differences in infection protocols between cell types limit direct statistical comparison. These findings align with previous reports showing higher titers in Calu-3 for various influenza strains [33,47] and lower susceptibility of A549 cells to some influenza A viruses [48], with viral replication in A549 improving at higher passage numbers [33]. Production of influenza virus stocks enables the study of viral infection in the laboratory. These stocks may contain both viruses and EVs, which are difficult to separate due to their similar biophysical properties [49,50]. Influenza virions measure 80–120 nm, comparable to exosomes’ size [50,51]. Ultracentrifugation is a widely used method for purifying both EVs and viruses. To characterize the components of different EV subpopulations, viral productions were subjected to differential ultracentrifugation at 3000× *g*, 17,000× *g*, and 100,000× *g*, isolating the 3K, 17K, and 100K fractions, respectively [26], which were then analyzed by DLS [22,52]. Analysis of the fractions identified three main nanoparticle subtypes: large, medium, and small, corresponding to size ranges generally reported for apoptotic bodies, microvesicles, and exosomes [12,16,43]. As shown by our results, ultracentrifugation pellets may contain mixed EV subpopulations due to size overlaps, making size-based separation alone unreliable [12,53,54]. Consequently, EV subtypes in our fractions cannot be formally classified as apoptotic bodies, microvesicles, or exosomes, since biogenesis mechanisms were not determined, following ISEV recommendations [13,16]. Biogenesis remains the primary distinguishing criterion among EVs. To improve isolation and characterization, ultracentrifugation should be combined with other purification methods, such as density or velocity gradients [10,16,52,55], which help reduce subpopulation overlap and remove non-vesicular contaminants while preserving particle integrity [55]. Size-exclusion chromatography can efficiently remove soluble protein contaminants while maintaining EV integrity [56], and immunoaffinity approaches targeting membrane markers such as CD9, CD63, or CD81 can enrich for specific EV subpopulations [13,22]. Although ultracentrifugation cannot fully separate viruses from EVs, it remains a useful approach for obtaining fractions enriched in particles within expected EV size ranges. Furthermore, DLS-based nanoparticle quantification revealed higher relative EV abundance in 17K or 100K pellets, depending on whether Calu-3 or A549 cells were used, and lower levels in 3K fractions. These findings suggest that EVs and viruses of similar sizes are more enriched in the 17K and 100K fractions. However, DLS provides reliable data only for homogeneous, non-aggregated particle populations [16], which may limit interpretation for complex samples such as 3K fractions containing large particles, as they include both EVs and complete or incomplete virions. Furthermore, differences in EV and viral protein profiles were observed between Calu-3 and A549 cells, reflecting the influence of cellular origin on the composition of co-purified EV/virion populations. Calu-3 cells, derived from bronchial epithelium, and A549 cells, derived from alveolar epithelium, display distinct structural and functional properties that may account for the distribution patterns detected across fractions. These findings highlight the relevance of including multiple human respiratory epithelial models when characterizing vesicle-enriched fractions produced during influenza infection.

EVs can modulate host immune responses, suppress innate immune signaling pathways, and assist viruses by carrying viral RNAs, proteins, and microRNAs that promote infection spread [15,31]. They may also undergo functional changes that reduce immune responses [10]. To assess their potential role in viral infectivity, we analyzed their content and determined TCID_50_ and PFU titers. Viral titers were highest in 17K fractions, followed by 100K and then 3K, consistent with DLS results and indicating that infectious particles in viral stocks are more enriched in the 17K and 100K fractions. To better assess EV infectivity, it is essential to separate viruses from EVs within fractions, using additional purification methods as previously described. Analyzing 17K fractions could be especially relevant for understanding viral pathogenesis, complementing the more commonly studied small-particle fractions [9,10,57] as viruses co-purified with these EVs appear more infectious. Since EVs in 3K and 17K pellets were larger than influenza virions (80–120 nm) [50], one or more influenza particles may have been enclosed within them. Indeed, EVs from infected cells have been shown to carry viral proteins such as HA, NS1, NP, PA, PB1, PB2, and M1 [9,10,45,58] and other respiratory viral infections like SARS-CoV-2, entire virions have been observed within EVs by electron microscopy [59].

In the final part of this study, co-purified viral particles and EVs were analyzed for their molecular content by Dot blotting using protein markers. As noted in the introduction, EV subtypes are characterized by specific protein markers [60]. CD9, HSP70, and flotillin-2, known as EV-associated proteins, were detected. As anticipated, CD9 was abundant in 100K, while HSP70 was also present but less abundant; both were detected at varying levels in 17K and 3K fractions. Flotillin-2 was strongly detected in 17K and 100K fractions of influenza A particles, and in 3K and 17K fractions of the B strain. However, marker distribution alone could not fully distinguish EV subpopulations. Nevertheless, distinct EV profiles were observed depending on whether influenza A or B was produced. Analysis of viral protein markers, hemagglutinin and nucleoprotein, showed the highest levels in 100K fractions, followed by 17K. The higher viral titers in 17K suggest that EVs purified at this speed may contain more infectious viral particles. Overall, viral protein distribution varied by virus type and fraction, suggesting differences in viral protein association mechanisms, and possibly in how viral particles are released or incorporated into EVs, depending on the influenza virus strain and host cell type.

## 5. Conclusions

This study revealed distinct differences in the physical and molecular characteristics of the fractions obtained by differential ultracentrifugation, with larger co-purified particles predominating in the 3K fraction, smaller ones in the 100K fraction, and intermediate-sized EVs co-purifying with infectious particles mainly in the 17K fraction. Moreover, protein analysis further confirmed the presence of both viral and EV-associated proteins, especially in 17K and 100K, with an enrichment of influenza A proteins in 17K and 100K fractions, with a more even distribution for influenza B. These findings highlight that the production profiles of EVs co-purified with viruses differed depending on the virus type and the infected cell line. Altogether, they represent a valuable starting point and provide a foundation for future investigations using more specific isolation methods to obtain purer fractions, thereby enabling more precise molecular characterization, evaluation of infectious potential, and elucidation of the role of EVs in viral dissemination.

## Figures and Tables

**Table 1:** MOI (TCID_50_/cell) Applied for Cell Infection.

Virus and Cell Lines	A/Vic	A/Dar	B/Aus
Calu-3	0.08	0.01	0.01
A549	0.8	0.1	0.1

**Table 2:** Polydispersity Index of Pellet Fractions from Each Viral Production.

	Fractions	Calu-3	A549
Control	3K	0.31	0.38
	17K	0.27	0.28
	100K	0.24	0.35
A/Vic	3K	0.31	0.37
	17K	0.28	0.21
	100K	0.18	0.36
A/Dar	3K	0.25	0.53
	17K	0.24	0.26
	100K	0.26	0.41
B/Aus	3K	0.36	0.32
	17K	0.28	0.19
	100K	0.27	0.25

## Data Availability

The data presented in this study are included in this published article. Virus sequence information can be found here: gisaid.org.

## Supplementary Materials

The following supporting information can be downloaded at https://www.mdpi.com/article/10.3390/v17111470/s1, Figure S1: Infectivity (in PFU) of Influenza Particles Associated with Purified EV-Containing; Table S1: Optimization of MOI (TCID_50_/cell) and viral tiers of viral stocks.

## Abbreviations

3K	3000× *g*
17K	17,000× *g*
100K	100,000× *g*
A/Vic	A/Victoria/4897/2022 (H1N1)
A/Dar	A/Darwin/9/2021 (H3N2)
B/Aus	B/Austria/1359417/2021 (B/Victoria)
DLS	Dynamic Light Scattering
EVs	Extracellular vesicles
HA	Hemagglutinin
ISEV	International Society for Extracellular Vesicles
MOI	Multiplicity of infection
NA	Neuraminidase
NP	Nucleoprotein
PDI	Polydispersity index
PFU	Plaque-forming units
TCID_50_	50% Tissue Culture Infectious Dose
TV	Total volume
