# Supplementary material for: Comparative Analysis of Extracellular Vesicle and Virus Co-Purified Fractions Produced by Contemporary Influenza A and B Viruses in Different Human Cell Lines
Source: Viruses. 2025 Nov 4;17(11):1470. doi: 10.3390/v17111470 (PMC12656816; doi:10.3390/v17111470)
Supplement: Supplementary file 1 [file viruses-17-01470-s001.zip › viruses-3908818-supplementary.pdf]

Supplementary Materials

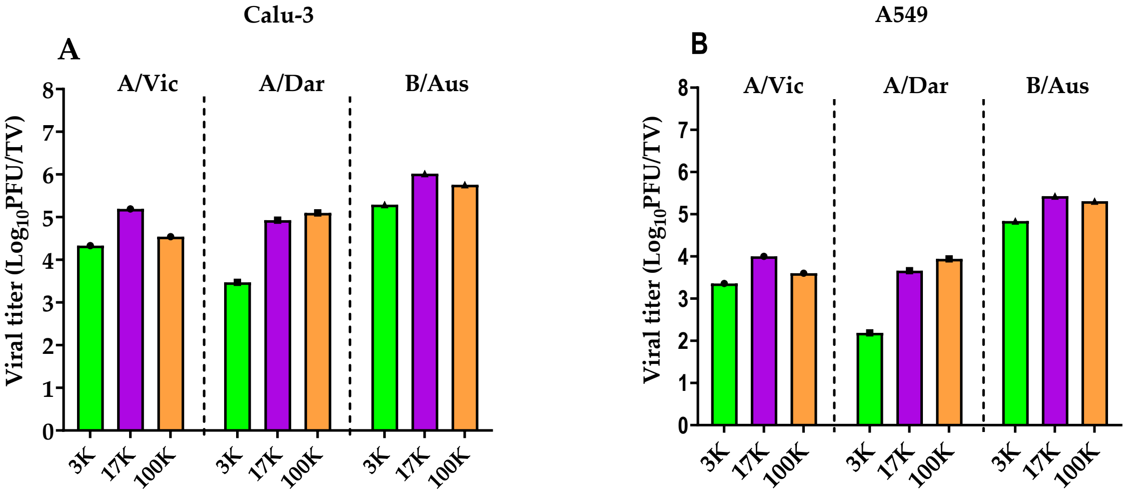

Figure S1: **Infectivity (in PFU) of Influenza Particles Associated with Purified EV-Containing Fractions.** A and B show PFU titers per total volume (TV) for A/Vic, A/Dar, and B/Aus viruses produced in (A) Calu-3 and (B) A549 cells, respectively. PFU values represent the mean of three technical replicates from a single biological sample. Graphs were generated using GraphPad Prism version 9.5.1. Statistical analysis was performed using two-way ANOVA. **** $p < 0.0001$; *** $p < 0.001$; ** $p < 0.01$; * $p < 0.05$.

Table S1: Optimization of MOI ($TCID_{50}$/cell) and viral tiers of viral stocks

Cells	Virus strain	MOI_1	Viral $titer_1$ ($log_{10}TCID_{50}$/mL)	MOI_2	Viral $titer_2$ (log_{10} $TCID_{50}$/mL)
Calu-3	A/Vic	0.04	2.2	0.4	5.075
	A/Dar	0.005	2.45	0.01	4.325
	B/Aus	0.007	5.2	0.01	5.575
A549	A/Vic	0.04	Under DL	0.3	3.825
	A/Dar	0.005	Under DL	0.05	2.325
	B/Aus	0.007	Under DL	0.07	4.45

Before producing viruses in Calu-3 and A549 cells, viral stocks of each virus, purchased from the National Institute for Biological Standards and Control (NIBSC, United Kingdom), were first amplified in MDCK cells overexpressing the α2.6 sialic acid receptor (MDCK α2,6). Viral titers were determined using the 50% tissue culture infectious dose ($TCID_{50}$) method, and mean titers were 7.95 log_{10} $TCID_{50}$/mL for A/Vic, 7.075 log_{10} $TCID_{50}$/mL for A/Dar, and 7.2 log_{10} $TCID_{50}$/mL for B/Aus. Before selecting the viral stock presented in the Results section, both Calu-3 and A549 cells were infected at adjusted MOIs to obtain titers suitable for our study. Table S2 summarizes the preliminary MOIs and titers obtained. Our detection limit (DL) of viral titers by $TCID_{50}$ method assay is 1.2 log_{10} $TCID_{50}$/mL.
